# The efficacy of newly proposed gastric open peroral endoscopic myotomy (GO-POEM) in preventing post-endoscopic submucosal dissection stenosis: A comparison with non-GO-POEM group

**DOI:** 10.1097/MD.0000000000036755

**Published:** 2023-12-29

**Authors:** Bong Ju Cho, Won Dong Lee, Jae Sun Song, Min A. Yang, Byung Sun Kim, Sung Yeol Yang, Gum Mo Jung, Ji Woong Kim, Yong Keun Cho, Jin Woong Cho

**Affiliations:** a Department of Internal Medicine, Presbyterian Medical Center, Jeonju, Republic of Korea.

**Keywords:** endoscopic submucosal dissection, gastric open peroral endoscopic myotomy, pyloric stenosis

## Abstract

Extensive endoscopic submucosal dissection (ESD) for gastric adenoma or early cancer can lead to post-ESD stenosis. This may cause a decrease in quality of life and an increase in medical issues. Therefore, this study examined the safety and effectiveness of gastric open peroral endoscopic myotomy (GO-POEM) in preventing stenosis following ESD. A retrospective investigation was carried out on 31 patients who underwent gastric ESD for > 75% of the lumen in the antrum or pylorus at the Presbyterian Medical Center in Korea between December 2004 and October 2022. The patients were divided into GO-POEM (n = 11) and non-GO-POEM groups (n = 20). The average age of the 31 patients was 73.23 years, and 18 were male. There were no differences in age, sex, location, gross findings, or procedure time between the 2 groups. In the GO-POEM group, only 1 patient (9 %) developed stenosis, compared to 11 patients (55 %) in the control group (*P* = .02). Multivariate analysis showed that the GO-POEM group had a significantly lower risk of post-ESD stenosis (*P* < .05). Stenosis symptoms resolved with a single endoscopic balloon dilatation (EBD) in 1 patient in the GO-POEM group. In contrast, 5 of 11 patients with stenosis in the non-GO-POEM group required a median of 2 EBD sessions (range, 1–8). GO-POEM may be an effective and reliable method for preventing stenosis post extensive gastric ESD. Further investigations are necessary to establish its efficacy and safety.

## 1. Introduction

Endoscopic submucosal dissection (ESD) is now widely recognized as the standard treatment for gastric adenoma and early cancer without lymph node metastasis.^[1–3]^ It is a minimally invasive procedure with good clinical outcomes.^[4,5]^

However, patients with extensive ESD, more than 75% of the circumference of the cardia, antrum, or pylorus are exposed to post-ESD stenosis.^[6,7]^ Although post-ESD stenosis is less common than other complications, such as bleeding or perforation, its management can be complex.^[8–10]^ Endoscopic balloon dilatation (EBD), local steroid injection, or oral steroid administration can help prevent post-ESD stenosis. Nevertheless, there is currently no established standard approach.^[11–13]^

Post-ESD stenosis can cause persistent symptoms such as dyspepsia, nausea, and vomiting, negatively impacting a patient’s quality of life.^[6]^ A recent study introduced a novel technique called gastric open peroral endoscopic myotomy (GO-POEM), which was safe and effective for preventing post-ESD stenosis.^[14]^ Therefore, this study focused on comparing the effectiveness of GO-POEM with that of non-GO-POEM in preventing post-ESD stenosis in patients who had undergone extensive gastric ESD.

## 2. Materials and methods

### 2.1. Patient selection

**Figure 1.**
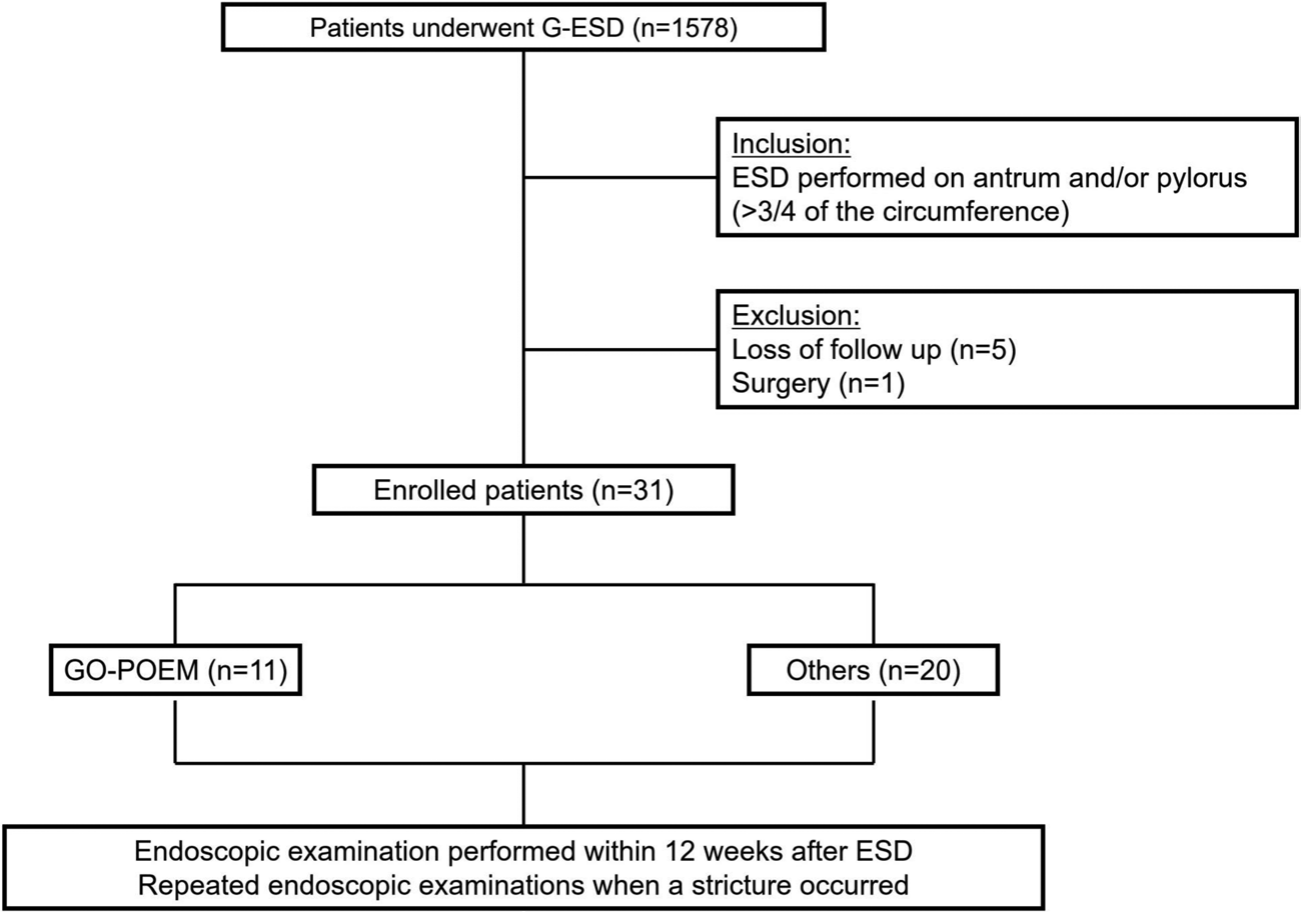
Flowchart showing inclusion and exclusion criteria of the study group. G-ESD = gastric endoscopic submucosal dissection; GO-POEM = gastric open peroral endoscopic myotomy.

We retrospectively reviewed the medical records of 4168 patients who underwent ESD for gastric adenoma or early gastric cancer at Presbyterian Medical Center between December 2004 and October 2022. Of the total patients, 37 underwent ESD on more than 3/4th of the circumference of the antrum or pylorus. Five patients who were not followed-up within 12 weeks after ESD and one who underwent additional surgery after noncurative ESD were excluded. As a result, the study comprised 31 patients, among whom 11 lesions underwent GO-POEM either during or after ESD (Fig. 1).

The Institutional Review Board of the Presbyterian Medical Center approved this study. Informed consent was waived due to the retrospective nature of the chart review (No. 2022-09-016).

### 2.2. ESD and GO-POEM

**Figure 2.**
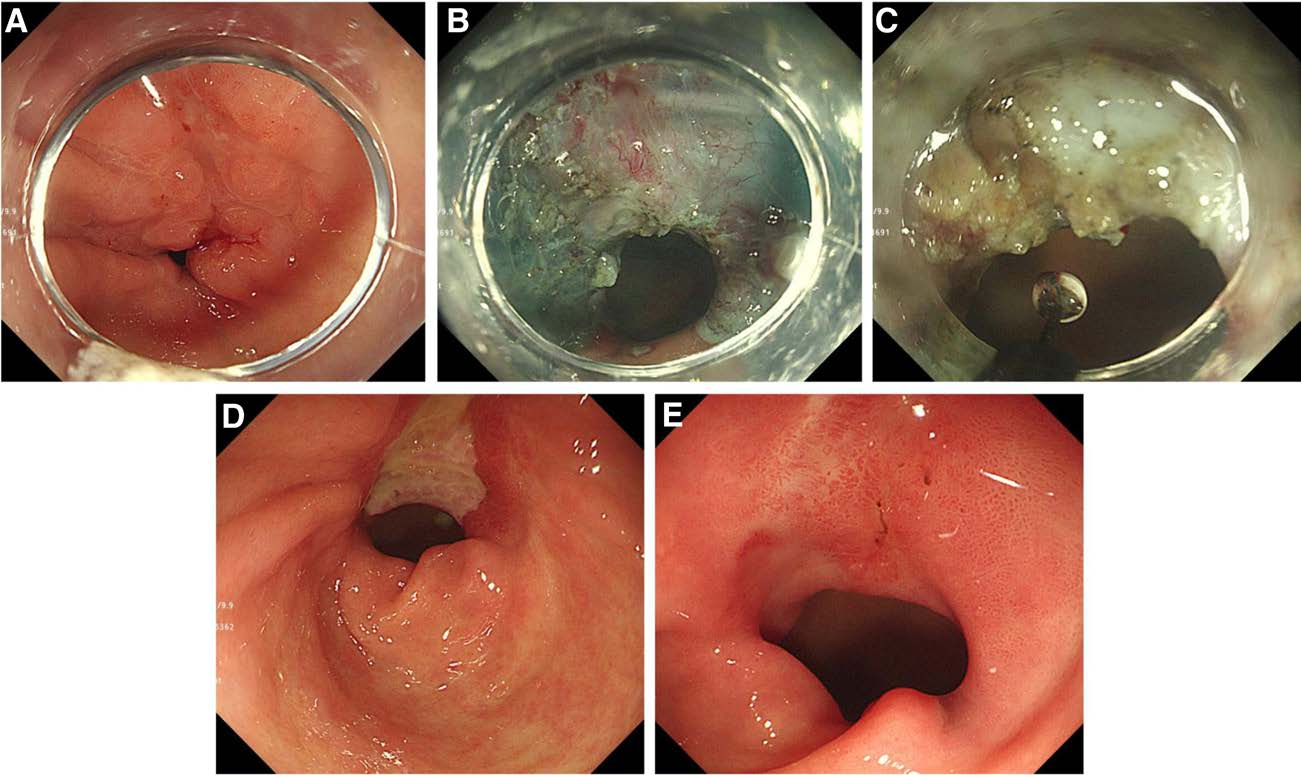
A case demonstrating the prevention of post-endoscopic submucosal dissection (ESD) stenosis after gastric open peroral endoscopic myotomy (GO-POEM) of the pylorus. (A), An adenoma was identified in the pyloric ring. (B), ESD was performed, resulting in the excision of 3/4 of the pyloric lumen. (C), A partial circular myotomy was performed. (D–E), Endoscopic follow-up was conducted at 2 months and 4 months, respectively.

A skilled endoscopist carried out all ESD procedures.^[15]^ Conscious sedation was achieved with midazolam and propofol during the procedures, which were performed using an endoscope (GIF-HQ290 or GIF-H260; Olympus, Tokyo, Japan) with a transparent cap (FM-EC0002; Finemedix, Daegu, Korea). A hook knife (KD-620LR; Olympus, Tokyo, Japan), an I-type knife (FM-EK0003-2; Finemedix, Daegu, Korea), an insulated-tip knife (IT) 2 (KD-611L; Olympus), and an electrosurgical generator (ERBE ICC 200; Tübingen, Germany) were used for submucosal dissection (Fig. 2A and B).

Immediately after or during the ESD procedure, the patients underwent partial myotomy (GO-POEM) with either an IT knife 2 or a Hook knife, with a cut current (Effect 2, 60 W). The myotomy was performed on the inner circular muscle from the oral to the anal side. If the ESD was carried out at the antral lesion, the cut was made at the center of the post-ESD site until a significant decrease in muscle contraction was observed. Using a Hook knife and directly visualizing the muscle layer with an endoscope, the myotomy reached approximately 2.5 to 3 cm in length. In cases where the ESD was conducted on the pyloric lesion, a straight myotomy of about 1 to 1.5 cm was performed using IT knife 2. The procedure was considered complete when there was an absence of pyloric contraction (Fig. 2C). To avoid perforation, we did not cut the longitudinal muscle.

### 2.3. Measurement of results

Baseline characteristics, including patient demographics such as sex and age, and the presence of diabetes and hypertension were compared in the study population. Lesion characteristics such as location, gross findings, longitudinal resection length, lesion ulcers, and margin involvement, were also compared. In addition, the study evaluated the pathological findings, use of oral steroids, and procedural time.

The location factor was divided into the pylorus and antrum. The endoscopic appearance was classified according to the Paris classification.^[16]^ We divided the patients into the flat type (IIb), elevated type (I, IIa, IIa + IIc), and depressed type (IIc, IIc + IIa, III). Post-ESD stenosis was characterized as the failure of a standard endoscope to pass through the antrum or pyloric ring. It was evaluated when ulcer scarring occurred at the ESD site.

### 2.4. Post-procedural management

Patients were scheduled for follow-up endoscopy between 4 and 12 weeks after ESD. The interval for the next follow-up endoscopy was determined on the basis of individual circumstances (Fig. 2D and E). Treatment was considered complete when ulcer scars were present at the ESD site or when the patient showed improvement in symptoms associated with post-ESD stenosis. Patients who were given oral steroids were gradually tapered off over 7 weeks. EBD was performed when conservative treatment did not improve obstructive symptoms.

EBD was carried out using a controlled radial expansion (CRE) balloon dilator (CRE Wire-Guided Balloon Dilation Catheter; Boston Scientific, Cork, Ireland) with adjustable diameters of 10 to 12 mm, 12 to 15 mm, and 15 to 18 mm for dilation. Under fluoroscopic guidance, a 0.035-inch hydrophilic guide wire was inserted across the stenotic pyloric canal and into the duodenum. The balloon was visually guided through the scope into the pylorus, positioned across the stricture. The balloon was slowly inflated using an insufflator with 3 to 8 atmospheres for 30 to 60 seconds. The inflation process was repeated 1 to 3 times with 1-minute intervals in 1 session. The endoscopist determined the balloon diameter and the number of EBD sessions based on the patient’s condition. The primary aim was to ease the passage of a standard endoscope through the stenotic lumen without resistance, thereby alleviating clinical symptoms.

### 2.5. Statistics

The baseline characteristics were compared between the GO-POEM and control groups. Possible confounding factors of post-ESD stenosis were compared between the stenotic and non-stenotic groups.

An independent t-test was used to analyze continuous variables. The data not normally distributed were analyzed using the Mann–Whitney *U* test. Independence of the 2 groups was compared using the chi-squared test for categorical variables. Fisher’s exact test was used when more than 20% of the cells had an expected frequency of 5 or less. Multivariate analysis using a logistic regression model was performed to analyze factors related to post-ESD stenosis.

Statistical significance was determined when the *P* value was < .05 of bivariate. All statistical analyses were performed using the IBM SPSS Statistics version 29 (IBM, Chicago, IL).

## 3. Results

### 3.1. General characteristics

Thirty-one patients who received ESD on lesions occupying greater than 3/4th of the lumen in the antrum or pylorus were included. The average age was 73.23 years, and 18 were male (58.06%), with no significant gender differences. Most of the endoscopic appearances were elevated type (96.77%). Eight patients (25.81%) were diagnosed with malignant tumors without submucosal invasion. The mean longitudinal resection size was 4.36 cm, with a procedure time of 49.45 minutes. Fifteen patients (48.39%) received oral steroids postoperatively.

The patients were divided into 2 categories depending on whether or not they underwent GO-POEM. No significant differences were noted in the general characteristics between the 2 groups (Table 1).

**Table 1:** Baseline information of the 2 groups.

	Total	GO-POEM	Control	*P* value
	N = 31	N = 11	N = 20	
Patient factor				
Sex (male, n, %)	18 (58.06)	6 (54.55)	12 (60)	1.000
Age (mean ± SD, year)	73.23 ± 9.03	74.27 ± 5.12	72.65 ± 10.67	.573*
Diabetes (n, %)	7 (22.58)	2 (18.18)	5 (25)	1.000
Hypertension (n, %)	16 (51.61)	4 (36.36)	12 (60)	.208†
Lesion factor				
Location (n, %)				1.000
Pylorus	21 (67.74)	8 (72.73)	13 (65)	
Antrum	10 (32.26)	3 (27.27)	7 (35)	
Ulcer (n, %)				1.000
Absent	24 (77.42)	9 (81.82)	15 (75)	
Present	7 (22.58)	2 (18.18)	5 (25)	
Pathology (n, %)				.676
Benign	23 (74.19)	9 (81.82)	14 (70)	
Malignant	8 (25.81)	2 (18.18)	6 (30)	
Lesion Type (n, %)				1.000
Flat	0	0	0	
Depressed	1 (3.23)	0	1 (5)	
Elevated	30 (96.77)	11 (100)	19 (95)	
Longitudinal resection length (mean ± SD, cm)	4.36 ± 1.53	4.18 ± 1.40	4.46 ± 1.62	.515
Margin positive (n, %)				.317
Positive	5 (16.13)	3 (27.27)	2 (10)	
Negative	26 (83.87)	8 (72.73)	18 (90)	
Procedure factor				
Total procedure time (mean ± SD, minutes)	49.45 ± 30.52	44.82 ± 18.07	52.00 ± 35.78	.885

Fisher’s exact test was used for categorical variables and the Mann–Whitney *U* test was used for continuous variables.

GO-POEM = gastric open peroral endoscopic myotomy, SD = standard deviation.

* Independent *t* test.

† Chi-square test.

### 3.2. Risk factors of post-ESD stenosis

12 out of 31 patients experienced stenosis after ESD. A comparison between the groups with and without stenosis was analyzed. There is no significant risk factor between the 2 groups except GO-POEM. Only 1 patient (9%) in the GO-POEM group developed stenosis, compared to 11 patients (55%) in the control group (*P* = .02) (Table 2).

**Table 2:** Risk factors of post-ESD stenosis.

	Stenosis		*P* value
	Present (n = 12)	Absent (n = 19)	
Patient factor			
Sex (male, n, %)	7(58.33)	11(57.89)	.981†
Age (yr) (mean ± SD, year)	76.42 ± 8.949	71.21 ± 8.709	.119‡
Diabetes (n, %)	3(25)	4(21.05)	1.000
Hypertension (n, %)	6(50)	10(52.63)	.886†
Lesion factor			
Location (n, %)			1.000
Pylorus	8(66.67)	13(68.42)	
Antrum	4(33.33)	6(31.58)	
Ulcer (n, %)			.676
Absent	10(83.33)	14(73.68)	
Present	2(16.67)	5(26.32)	
Type (n, %)			.387
Flat	0	0	
Depressed	1(8.33)	0	
Elevated	11(91.67)	19(100)	
Margin (n, %)			1.000
Positive	2(16.67)	3(15.79)	
Negative	10(83.33)	16(84.21)	
Pathology (n, %)			.676
Benign	8(66.67)	15(78.95)	
Malignant	4(33.33)	4(21.05)	
Longitudinal resection length (mean ± SD, cm)	4.86 ± 1.89	4.04 ± 1.20	.087
Procedure factor			
GO-POEM (n, %)			.02*
Positive	1(8.33)	10(52.63)	
Negative	11(91.67)	9(47.37)	
Total procedure time (mean ± SD, minutes)	58.42 ± 37.15	43.79 ± 24.94	.123
Steroid (n, %)			.552
Use	5(41.67)	10(52.63)	
Nonuse	7(58.33)	9(47.37)	

Fisher’s exact test was used for categorical variables and the Mann–Whitney *U* test was used for continuous variables.

GO-POEM = gastric open peroral endoscopic myotomy, SD = standard deviation.

* *P* < .05.

† Chi-square test.

‡ Independent *t* test.

Logistic regression analysis was conducted considering longitudinal resection length (*P* = .087) and GO-POEM (*P* = .02). Post-ESD stenosis was significantly lower in the GO-POEM group (*P* < .05, OR 0.082) (Table 3).

**Table 3:** Multivariate logistic regression analysis of the compounding factors of post-ESD stenosis.

	Univariate			Multivariate		
	95% CI	OR	*P* value	95% CI	OR	*P* value
GO-POEM	0.09–0.766	0.082	.028*	0.09–0.766	0.082	.030*
Longitudinal resection length	0.854–2.499	1.461	.166	0.085–2.912	1.531	.195

CI = confidence interval, GO-POEM = gastric open peroral endoscopic myotomy, OR = odds ratio.

* *P* < .05.

### 3.3. Treatment outcomes for GO-POEM

Stenosis symptoms after ESD varied from mild indigestion to severe symptoms, such as nausea, vomiting, abdominal distension, or decreased oral intake. A conservative approach was adopted in patients with mild symptoms.

In the group that underwent GO-POEM, only 1 patient experienced post-ESD stenosis, which resolved with a single EBD. However, in the group without GO-POEM, 5 of 11 patients with stenosis required EBD. The median number of EBD cases was 2 (range, 1–8). No severe complications were observed in any of the patients in either group.

The median time to symptom onset was 65 days in the GO-POEM group and 51 days (range, 22–90) in the non-GO-POEM group. After treatment, the median time for symptom resolution was 34 days in the GO-POEM group and 84 days (range, 33–219) in the non-GO-POEM group. This showed that the GO-POEM group had a slower onset of symptoms, quicker resolution of symptoms, and required fewer sessions of EBD (Table 4).

**Table 4:** Comparison of the occurrence and duration of stenosis-related symptoms and endoscopic balloon dilatation requirements between the GO-POEM and control groups after the procedure.

	Stenosis		
	Total (N = 12)	GO-POEM (N = 1)	Control (N = 11)
Time to post-ESD stenosis (median, range, d)	51 (22–90)	65	51 (22–90)
Associated symptoms remission period (median, range, d)	76 (33–219)	34	84 (33–219)
Number of patients underwent EBD (n)	6	1	5
Number of EBD required (median, n, range)	2 (1–8)	1	2 (1–8)

EBD = endoscopic balloon dilation, ESD = endoscopic submucosal dissection, GO-POEM = gastric open peroral endoscopic myotomy.

Five patients including 2 patients in the GO-POEM group experienced postoperative bleeding after ESD, controlled with endoscopic treatment. No severe adverse events were recorded, such as perforation or massive rebleeding.

## 4. Discussion

Gastric post-ESD stenosis occurs due to the healing of ESD ulcers within several weeks and is experienced in 1.9% to 2.5% of gastric ESD cases.^[6,8–10,17–19]^ The risk of post-ESD stenosis is significant when there is a circumferential mucosal defect in > 75% of the antrum and cardia.^[6,7]^ Although some patients with stenosis may not exhibit symptoms, it is crucial to acknowledge the potential for a significant decline in the quality of life when symptoms do occur.

EBD, steroid administration, and temporary stent insertion have been investigated for the prevention of stenosis after extensive gastric ESD.^[6,11,20,21]^ EBD was effective in preventing stenosis when it was performed early post-ESD.^[12,22]^ Structural changes, thin mucosa, severe fibrosis, and the need for multiple EBD procedures are risk factors for perforation.^[19,23]^ An alternative preventive method involves the administration of local steroid injections and/or oral steroids. Steroids have been effectively used to prevent esophageal ESD stenosis, but their effect on gastric ESD remains unclear.^[11,21]^ Many studies assessing the effectiveness of oral prednisolone and triamcinolone injections had limited sample sizes.^[21,24,25]^ Kishida et al^[11]^ observed no significant difference in the occurrence of stenosis between the steroid and non-steroid groups in 107 patients. Temporary stent insertion has also been attempted for post-ESD stenosis management, but stent migration remains challenging.^[20]^

GO-POEM was specifically designed to prevent stenotic symptoms and was introduced by Lee et al^[14]^ as a novel technique for preventing stenosis post-ESD. The safety and efficacy of GO-POEM were demonstrated in a case study involving 10 patients. Treatment works by reducing contractions of the antrum and pylorus through circular myotomy during or after the ESD procedure, specifically for patients with a high risk of post-ESD stenosis. Circular myotomy slows the healing of the artificial ulcer by preventing contraction.^[26]^ In addition, it increases the diameter of the pylorus, as reported in a study on treating gastroparesis.^[27]^

This was a retrospective comparative study on the efficacy of GO-POEM. The incidence of post-ESD stenosis was lower in the GO-POEM group. The procedure was quick and simple to perform during or after ESD without additional courses, with a procedure time of 4 minutes (range, 2–16 minutes). No complications were associated with the GO-POEM.

Post-ESD stenosis in pre-pyloric lesions generally requires more balloon dilatation and a longer treatment period than cardiac lesions. Coda et al reported that the median number of EBD required for pre-pylorus stenosis was 9 times (range, 7–40), and the median treatment period was 50 days (range, 28–190).^[6]^ As per our study, stenosis in the GO-POEM group required only one EBD for improvement, compared to a maximum of 8 EBDs in the control group (median, 2; range, 1–8). The EBD treatment period was shorter in the GO-POEM group (34 days) than in the control group (84 days). GO-POEM decreases the frequency of repeat EBD and shortens the treatment period in patients with stenosis, reducing the time and cost of treatment.

Our study had the following limitations. First, the data were collected retrospectively from medical records at a single center, including a small number of post-ESD stenosis cases. This may have resulted in a selection bias and limited detailed subgroup analyses. Second, oral steroids were administered to 15 patients in both the GO-POEM and non-GO-POEM groups. Although the influence of steroids on gastric stenosis remains uncertain, their potential impact on the outcome should be considered. Third, it is challenging to generalize the results because an endoscopy expert conducted all procedures at a single center.

This is the first study comparing the safety and effectiveness of the newly proposed prophylactic GO-POEM with a control group in high-risk ESD stenosis patients. GO-POEM is an effective and safe method for preventing post-ESD stenosis in the antrum and pylorus. A large-scale multicenter study is required to determine the optimal management approach and provide an accurate assessment of the long-term effects of GO-POEM.

## Acknowledgments

We would like to thank all the staff and nurses at the gastrointestinal endoscopy center at Presbyterian Medical Center.

## Author contributions

**Conceptualization:** Jin Woong Cho.

**Data curation:** Byung Sun Kim, Gum Mo Jung, Ji Woong Kim, Yong Keun Cho.

**Formal analysis:** Won Dong Lee, Jae Sun Song, Min A Yang, Sung Yeol Yang.

**Methodology:** Jin Woong Cho.

**Writing – original draft:** Bong Ju Cho, Jin Woong Cho.

**Writing – review & editing:** Bong Ju Cho, Jin Woong Cho.
